# Macrophage traits in cancer cells are induced by macrophage-cancer cell fusion and cannot be explained by cellular interaction

**DOI:** 10.1186/s12885-015-1935-0

**Published:** 2015-11-20

**Authors:** Ivan Shabo, Kristine Midtbö, Henrik Andersson, Emma Åkerlund, Hans Olsson, Pia Wegman, Cecilia Gunnarsson, Annelie Lindström

**Affiliations:** 1 Division of Surgery, Department of Clinical and Experimental Medicine, Faculty of Health Science, Linköping University, SE 581 85, Linköping, Sweden; 2 Division of Cell Biology, Department of Clinical and Experimental Medicine, Faculty of Health Science, Linköping University, Linköping, Sweden; 3 Division of Clinical Genetics, Department of Clinical and Experimental Medicine, Faculty of Health Science, Linköping University, Linköping, Sweden; 4 Division of Pathology, Department of Clinical and Experimental Medicine, Faculty of Health Science, Linköping University, Linköping, Sweden; 5 Department of Surgery, County Council of Östergötland, Linköping, Sweden; 6 Endocrine and Sarcoma Surgery Unit, Department of Molecular Medicine and Surgery, Karolinska Institutet, Stockholm, Sweden; 7 Department of Breast and Endocrine Surgery, Karolinska University Hospital, SE 171 77, Stockholm, Sweden

**Keywords:** Cell fusion, Macrophages, Paracrine cellular interaction, Tumor markers

## Abstract

**Background:**

Cell fusion is a natural process in normal development and tissue regeneration. Fusion between cancer cells and macrophages generates metastatic hybrids with genetic and phenotypic characteristics from both maternal cells. However, there are no clinical markers for detecting cell fusion in clinical context. Macrophage-specific antigen CD163 expression in tumor cells is reported in breast and colorectal cancers and proposed being caused by macrophages-cancer cell fusion in tumor stroma. The purpose of this study is to examine the cell fusion process as a biological explanation for macrophage phenotype in breast.

**Methods:**

Monocytes, harvested from male blood donor, were activated to M2 macrophages and co-cultured in ThinCert transwell system with GFP-labeled MCF-7 cancer cells. MCF7/macrophage hybrids were generated by spontaneous cell fusion, isolated by fluorescence-activated cell sorting and confirmed by fluorescence microscopy, short tandem repeats analysis and flow cytometry. CD163 expression was evaluated in breast tumor samples material from 127 women by immunohistochemistry.

**Results:**

MCF-7/macrophage hybrids were generated spontaneously at average rate of 2 % and showed phenotypic and genetic traits from both maternal cells. CD163 expression in MCF-7 cells could not be induced by paracrine interaction with M2-activated macrophages. CD163 positive cancer cells in tumor sections grew in clonal collection and a cutoff point >25 % of positive cancer cells was significantly correlated to disease free and overall survival.

**Conclusions:**

In conclusion, macrophage traits in breast cancer might be caused by cell fusion rather than explained by paracrine cellular interaction. These data provide new insights into the role of cell fusion in breast cancer and contributes to the development of clinical markers to identify cell fusion.

## Background

The theory of cell fusion in cancer states that cancer cells may produce hybrids with metastatic phenotype due to spontaneous fusion with migratory leukocytes. The hybrids acquire genetic and phenotypic characteristics from both maternal cells [1, 2]. Somatic cells acquire nuclear reprogramming and epigenetic modifications to form pluripotent hybrid cells without any changes occurring to their nuclear DNA [3]. The direction of nuclear reprogramming is decided by the ratio of genetic material contributed by the maternal cells [4]. Thus, cell fusion is an efficient process of rapid phenotypic and functional evolution that produces cells with new properties at a much higher rate than random mutagenesis.

Several reports present evidence that macrophages are an important partner in this process. Fusion between macrophages and cancer cells generates hybrids with increased metastatic potential [5, 6]. Powell et al. in an experimental animal model with parabiosis, showed in vivo evidence of fusion between circulating bone-marrow-derived cells (BMDCs) and tumor epithelium during tumorigenesis, demonstrating that macrophages were a cellular partner in this process [7]. Silk et al. (2013) provided evidence that transplanted cells of the BMDCs incorporate into human intestinal epithelium through cell fusion [8]. Circulating hybrids are also reported in colorectal and pancreatic cancer patients [9].

Based on cell fusion theory and the assumption that the macrophage–cancer cell fusion creates hybrids expressing phenotypic characteristics of macrophages, we reported in previous studies that the macrophage-specific marker, CD163, was expressed in breast and colorectal cancers. CD163 expression in cancer cells was significantly related to advanced tumor stages and poor survival [10, 11]. Fusion events in human cancers are difficult to detect in a clinical context. Clinically, it is difficult to confirm that CD163 expression in tumor tissue is caused by cell fusion because the genetic content of macrophages, cancer cells and any hybrids have the same origin. Further, the expression of CD163 in cancer cells could be explained by other biological processes like abnormal phenotypic expression in cancer cells and paracrine cellular interaction between cancer cells and macrophages [12, 13]. To study the clinical significance of cell fusion in breast cancer, it is important to identify specific markers for this process in clinical tumor material.

In the present study, we have designed an experimental model where the presence of macrophage phenotype in breast cancer cells is examined on the basis of the previously mentioned arguments. Here we review data that CD163 expression is caused by cell fusion and not induced by paracrine cellular interaction.

## Methods

### Cell culture

MCF-7/GFP breast cancer cell line (Cell Biolabs, INC. San Diego, USA) was cultured in Roswell Park Memorial Institute (RPMI) 1640 medium supplemented with 1 % PEST, 10 % FBS, 2.5 % HEPES and 1 % L-glutamine (Gibco®, Life Technologies, USA) in a T-75 tissue culture flasks (Sigma-Aldrich Co, ST. Louis, USA) and incubated at 37 °C in humidified air 5 % CO_2_ atmosphere. Cell medium was changed every 2–3 days, and the cells were passaged at 95 % confluence.

### Monocyte isolation

Monocytes were isolated from buffy coat obtained from male healthy blood donors at the department of Transfusion Medicine, County Council of Östergötland, in Linköping, Sweden. All the blood donors had given their informed consent according to the local guidelines (University Hospital in Linköping) and the Swedish National Law on ethical review of research involving humans (2003:460: 3–4 §). The buffy coat was mixed with 70 ml NaCl, layered onto Lymphoprep (Axis-Shield, Oslo) and centrifuged at 480 g in room temperature for 40 minutes. The mononuclear cell layer was collected into new tubes and washed twice with PBS-Heparin for 5 min and centrifuged at 220 g in 4 °C. The white blood cells were seeded to T-75 tissue culture flasks with RPMI 1640 medium, supplemented with 1 U/ml penicillin, 10 μg/ml streptomycin and incubated for 1–2 h to allow monocyte adhesion. The non-adherent cells were eliminated by washing 2–3 times using PBS. The adherent monocytes were allowed to differentiate to macrophages with 40 ng/ml of macrophage colony-stimulating factor, M-CSF (Nordic Biosite, Sweden) for 5–7 days. To induce M2 macrophages, the M-CSF differentiated macrophages were stimulated with 20 ng/ml human interleukin-4, IL-4 (Nordic Biosite, Sweden) for 18–24 h.

### Cell fusion and cellular interaction model

**Fig. 1:**
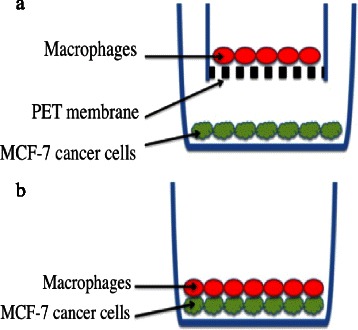
Transwell culture system. The porous bottom of the insert provides independent access to both sides of a cell monolayer, allowing in vitro cellular interactions (**a**). Spontaneous cell fusion was allowed by culturing MCF-7 cancer cells and M2 macrophages along the bottom of the same chamber (**b**)

Green fluorescent protein (GFP) labeled MCF-7 cancer cells and macrophages were co-cultured in ThinCert^TM^ cell culture inserts (Greiner Bio One, Kremsmünster, Austeria), where both cell types were allowed to have cellular interaction without physical contact to prevent cell fusion. The macrophages (5x10^5^) were seeded in the upper chamber on a polyethylene terephthalate (PET) membrane with 0.4 μm pores and physically separated from MCF-7/GFP cancer cells that cultured in bottom of the lower chamber. This cell culture model allows intercellular signaling via e.g. cytokines and exosomes, which can freely pass through the PET-membrane pores between the cells (Fig. 1a). It does not allow cell fusion.

To induce spontaneous cell fusion, macrophages and GFP-labeled MCF-7 cancer cells were co-cultured in the same cell culture vial in RPMI 1640 medium during 2–3 days. The cells were seeded at a ratio of about 3–5:1 (macrophages: MCF-7). Cell fusion experiments were repeated several times, and approximately 5x10^5^ macrophages were used in each trial. We estimated the size of the population of hybrids on the basis of the number of macrophages cultured with MCF-7 cancer cells. We did so for a number of reasons, viz. MCF-7 cancer cells proliferate rapidly, macrophages do not undergo cell division, and we assumed that a hybrid cell is generated by fusion between a macrophage and a cancer cell (Fig. 1b).

### Fluorescence-activated cell sorting (FACS)

Cells were washed once with PBS and harvested with a 0.05 % trypsin-EDTA solution. Detached cells were washed with PBS and resuspended in 95 μl Cell Staining Buffer (Biolegend, San Diego, USA) at a concentration of about 5x10^6^ cells/ml. The cell suspension was incubated on ice for 10 min with 5 μl TrueStain FcX solution (BioLegend, San Diego, USA) per 1x10^6^ cells. Combinations of direct conjugated monoclonal anti-human CD163 (APC Anti-human CD163 (IgG1 k), clone GHI/61, con 100 μg/ml) and anti-human CD45 (PerCP/Cy5.5 anti-human CD45 (IgG1 k), clone HI30, 50 μg/ml) antibodies or their respective isotype controls (APC and PerCP/Cy5.5 mouse IgG1 k, clone MOPC-21, con 200 μg/ml) (Biolegend, San Diego, USA) were added to the cell suspension at concentrations recommended by the manufacturer and incubated at 4 °C in the dark for 30 min. The labeled cells were washed twice and diluted in 1 ml PBS and filtrated in pre-separation filter 30 μm (Miltenyi Biotech, Lund, Sweden) before flow cytometry analysis. Cells in both the ThinCert culture system and co-culture were examined initially with a Gallios flow cytometer (Beckman Coulter, Inc.) and cells were sorted with BD FACSAria™ III (BD Bioscience, USA). The cells were examined in relation to GFP, CD163 and CD45 expression. Cells were initially sorted by GFP expression (positive selection of MCF-7/GFP origin) and subsequently by CD163 and CD45 expression (positive selection of cancer cells with macrophage phenotype).

### Immunofluorescence microscopy

Macrophages, MCF-7 cells and hybrids (1x10^5^ cells) were seeded on coverslips and incubated 24 h in RPMI + 10 % FBS. Cells were fixed with 4 % paraformaldehyde for 30 min at 37 °C, washed once in PBS followed by permeabilization/blocking for 30 min in 2 % BSA/0.1 % Saponin in PBS. Cells were then incubated with a mouse monoclonal α-CD163 antibody (Abcam) in PBS/0.5 % BSA for 2 h at room temperature and washed three times with PBS. A secondary antibody goat anti-mouse IgG Alexa Fluor 546 (Invitrogen) was added in PBS/0.5 % BSA for 45 min, followed by three washes with PBS. The cover slips were mounted on microscope slides in Dako fluorescence mount media containg DAPI. Fluorescence images were taken with a Zeiss Axiovert 200 M fluorescence microscope with a Zeiss Plan-APOCHROMAT 63x/1.4 oil DIC objective.

### Immunostaining and expression levels of CD163 in relation to survival data

To investigate whether the proportions of CD163 positive breast cancer cells have been correlated to clinical data, we re-evaluated breast cancer specimens from 127 women, a well controlled patient material that was reported in previous studies [11, 14, 15]. Written informed consent for participation in research was obtained from participants in connection with previous studies. Ethical approval from the Regional Ethics Committee in Linköping obtained according to Swedish Biobank Law (Reference number: 2010/311–31). The patients were diagnosed and treated using conventional methods at surgical departments in southeastern Sweden. All patients were in Stage II according to the UICC, and all received adjuvant tamoxifen therapy. These specimens had previously been collected in a tissue microarray and originated from a Swedish randomized trial of 2 versus 5 years of tamoxifen treatment. Serial sections of 5 mm were cut from tissue array blocks, deparaffinized in xylene, and hydrated in a series of graded alcohols (100 %, 95 %, and 70 %). Heat-induced antigen retrieval was carried out using a water bath pretreatment in Tris Ethylenediaminetetraacetic acid (1 mM, pH 9) for 50 min before staining for CD163. Detection was carried out using the DAKO Envision system. The immunoreactivity of CD163 was characterized by granular cytoplasmic, or cytoplasmatic and membrane staining patterns. In negative control samples, the primary antibody was replaced by an isotype-antimouse immunoglobulin G1 antibody. All immunostaining was evaluated by two of the authors (HO and IS) and scored on a 5-tiered score as follows: 0 %, 1–25 %, 26–50 %, 51–75 %, and 76–100 % of the cancer cells. Macrophages and cancer cells could be distinguished on morphological basis. Macrophage nuclei were small and regular, whereas the cancer cells were enlarged and atypical with pleomorphic hypertrophic and darker nuclei. Moreover, cancer cells show a decreased cytoplasmic - nuclear ratio.

To investigate the significance of CD163 expression levels in relation to survival data, we used four different cut-off points 1–25 %, 25–50 %, 50–75 % and 57–100 % of CD163 positive cancer cells in tumor sections. The correlation of CD163 expression levels and survival rates, both disease specific survival (DSS) and distant recurrence free survival (DRFS), was estimated using Kaplan-Meier analyses and the log rank test.

### STR analysis/Quantitative fluorescent PCR

DNA was extracted from macrophage, MCF-7 breast cancer cells and MCF-7/macrophage hybrid cell suspensions in a biorobot (EZ1, Qiagen) with DNA Tissue kit (Qiagen) following the manufacturer’s instructions. Each sample was then subjected to multiplex amplification of 24 STR markers on chromosomes 13, 18, 21, X and Y in two sets of tubes (Table 1) using ChromoQuant® QF PCR kit (Cybergene AB). The PCR was carried out in 25 μl reactions containing 14.6 μl mastermix, 0.4 μl GoTaq polymerase (Promega Inc) and 10 μl DNA (1.5 ng/μl). An initial denaturation at 94 °C for 3 min was followed by 26 cycles of 30 seconds at 94 °C, 1 min of annealing at 57 °C, and 2 min of extension at 71 °C. An extension period of 5 min at 71 °C followed the final cycle.

**Table 1:** Size and number of alleles from short tandem repeat (STR) analysis

STR marker	Hybrids	Macrophages	MCF7 cells
AMEL	105, 110/2	105, 110/2	105/1
DXYS218	323, 327, 331/3	327, 331/2	327/1
SRY	204/1	204/1	-
X22	205, 209, 224/3	205, 224/2	209/1
XHPRT	277, 289/2	277/1	289/1
DXS6803	123, 126/2	126/1	123/1
DXS6854	107/1	107/1	107/1
D13S305	449/1	445, 456/2	448/1
D13S628	455/1	456/1	455/1
D13S634	394, 399/2	394/1	399/1
D13S742	287/1	262, 290/2	286/1
D13S797	196, 200/2	196/1	196, 200/2
D18S386	351, 355, 388, 392/4	355, 388 /2	351, 392/2
D18S390	338, 342/2	342/1	338, 342/2
D18S391	160/1	160/1	160/1
D18S535	475, 483, 487/3	475, 487/2	483/1
D18S819	251, 254, 262/3	251, 254/2	262/1
D18S976	185/1	186, 189/2	185/1
D21S11	253, 267/2	245, 255/2	253, 267/2
D21S1246	293, 297, 318/3	293, 318/2	293, 297/2
D21S1409	203, 210, 214/3	210, 214/2	203, 214/2
D21S1411	307, 312/2	299, 332/2	307, 312/2
D21S1435	375, 383, 387/3	375, 383/2	375, 387/2
D21S1444	314, 318/2	318/1	314, 318/2

STR analysis was used to determine the DNA profile of M macrophages, GFP-labeled MCF-7 breast cancer cells, and MCF-7/macrophage hybrids. A total of 24 loci on X and Y chromosomes as well as chromosomes 13, 18 and 21 were used in this analysis. The macrophages originated from M2-activated monocytes harvested from male blood donors and contained Y chromosome. MCF-7 is a breast cancer cell line isolated originally from a female patient. Of 24 markers, 17 were of the same allelic size in MCF-7/macrophage hybrids. For several loci at least one allele was common to macrophages and MCF-7 cancer cells represented in the hybrids. Sex-determining region Y gene (SRY) is one of the markers found in MCF-7/macrophage hybrids, indicating that these cells originate from fusion between macrophages and MCF-7 cells. Note that the hybrids contain the same size of alleles on the X-chromosome markers as do macrophages and MCF-7 cells

PCR products were separated by capillary electrophoresis on an ABI 3130xl Genetic Analyzer (Applied Biosystems). For each well, 1 μl PCR product was mixed with 12 μl HiDi formamide and 0.3 μl GeneScan-500ROX size marker, followed by denaturation at 95 °C for 2 min before loading. The POP7 polymer was used in the electrophoresis, and results were analyzed using GeneMapper software version 4 (Applied Biosystems).

## Results

### MCF-7/macrophage hybrid cell generation and transwell co-culture of macrophages and GFP-labeled MCF-7 cancer cells

**Fig. 2:**
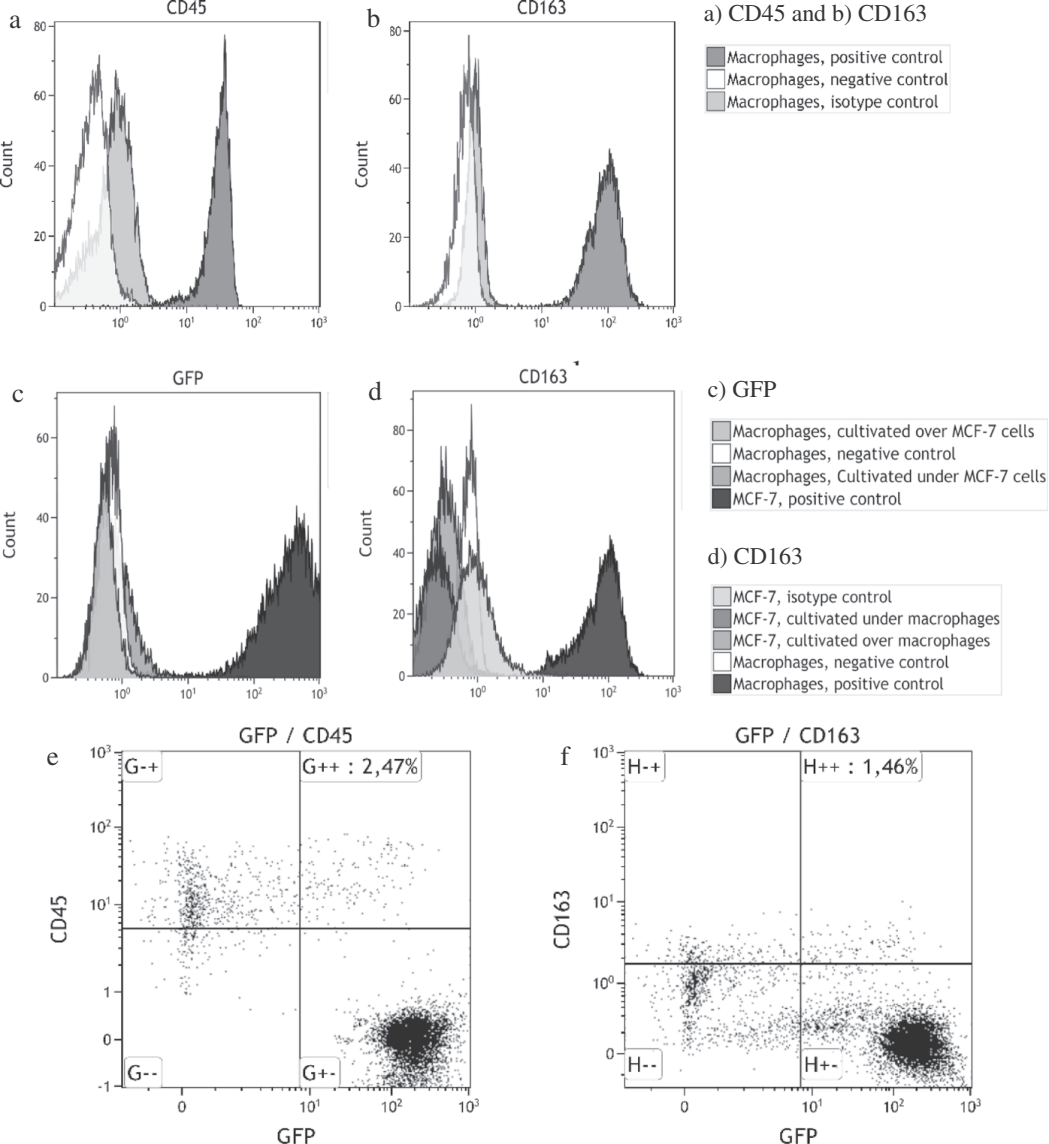
Flow cytometry analysis of M2 macrophages, GFP-labeled MCF-7 breast cancer cells and MCF-7/macrophage hybrids. **a**) M2 macrophages exhibit CD45 expression when stained with anti-CD45, showing an increase in fluorescence intensity compared to the isotype and negative controls. **b**) M2 macrophages exhibit CD163 expression when stained with anti-CD163, showing an increase in fluorescence intensity compared to the isotype and negative controls. **c**) The transfected MCF-7 cells exhibit GFP expression. The macrophages were not expressing GFP and no GFP were detected in macrophages after co-culture in ThinCert transwell culture system. Regardless if macrophages were cultivated over (upper chamber) or under (lower chamber) GFP-labeled MCF-7 cells in ThinCert transwell cuture system. In this transwell culture system the cells share culture medium, allowing paracrine signaling, but the cells are physically separated by a filter, preventing cellular contact and cell fusion. **d**) The MCF-7 cells did not express CD163, nor were CD163 expression induced after co-culture with macrophages in the ThinCert transwell culture system, indicating that macrophage traits in cancer cells were not generated by cellular interaction between macrophages and cancer cells. **e**) Co-cultured MCF-7 cells and macrophages, created hybrids by spontaneous cell fusion. The hybrids expressed phenotypic characteristics from maternal cells, GFP-labeled MCF-7 breast cancer cells and M2 macrophages. Note that the hybrids were identified by exhibiting a double positive phenotype, positive for green fluorescence protein GFP and macrophage-specific antigen CD163, or f) panleukocyte marker CD45

One approach to investigating whether cellular interaction between the macrophages and cancer cells can induce macrophage phenotype in cancer cells is to co-culture both cell types in a transwell culture chamber system. Macrophages and cancer cells (GFP-labeled MCF-7 cell line) were co-cultured in the same chamber but separated by a polyester membrane with 0.4 μm pores to allow cellular interaction without physical contact to prevent cell fusion (Figs. 1a, 2c-d). We also co-cultured macrophages and GFP-labeled MCF-7 cancer cells in the same chamber to create MCF-7/macrophage hybrids by spontaneous cell fusion (Figs. 1b, 2e-f).

MCF-7/macrophage hybrids were generated spontaneously after three days by co-culturing MCF-7 cancer cells with macrophages. The hybrids were defined as GFP^+^/CD163^+^/CD45^+^ positive cells and were separated by FACS. Cells that expressed only GFP were sorted as MCF-7 cancer cells, and GFP-negative cells were defined as macrophages. This experiment was repeated several times, and the proportion of hybrids averaged about 2 % in each experiment. Flow cytometry analysis showed that the GFP+ hybrids expressed both the macrophage-specific marker, CD163, and the leukocyte common antigen, CD45 (Fig. 2e-f).

As experimental controls MCF-7 cancer cells and macrophages co-cultured for three days in the same medium in a transwell chamber system were also analyzed by flow cytometry for GFP, CD163 and CD45 expression. Macrophages expressed both CD163 and CD45, but showed no GFP expression (Fig. 2c). The MCF-7 cancer cells clearly expressed GFP, but neither CD45 nor CD163 despite repeated transwell chamber system experiments (Fig. 2d). Even when the experiments were repeated with different durations (3, 5 and 7 days) of co-culture, the outcome remained the same (data not shown).

### Fluorescence microscopy

**Fig. 3:**
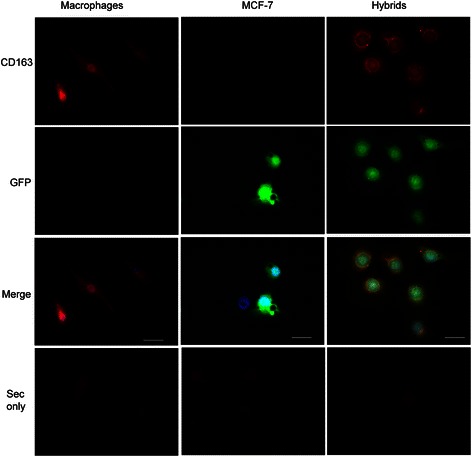
Fluorescence microscopy. Macrophages, MCF-7/GFP cells and MCF-7/macrophage hybrids, created by spontaneous cell fusion between GFP-labeled MCF-7 breast cancer cells and M2 macrophages were stained with an α-CD163 antibody and DAPI, and analyzed by fluorescence microscopy. Cells stained with secondary antibody only were used as negative control for CD163. The hybrids show cytoplasmatic expression of macrophage-specific antigen CD163 (red) and GFP, which are inherited traits from both maternal cells. Bars = 20 μm

The expression of GFP, CD45 and CD163 in all cell types was confirmed by fluorescence microscopy. Macrophages, MCF-7 and hybrids were seeded on glass cover slips and immunostained for CD163. The hybrids had inherited GFP expression from MCF-7 cells and CD163 expression from macrophages. From the transwell culture chamber system, the macrophages showed distinct expression of CD163 but not of GFP. The GFP-labeled MCF-7 cells showed no CD163 expression. The hybrids retained the MCF-7 cancer cell morphology, which is characterized by a large nucleus, irregular shape, and a small cytoplasmic amount (Fig. 3).

### DNA profiling

**Fig. 4:**
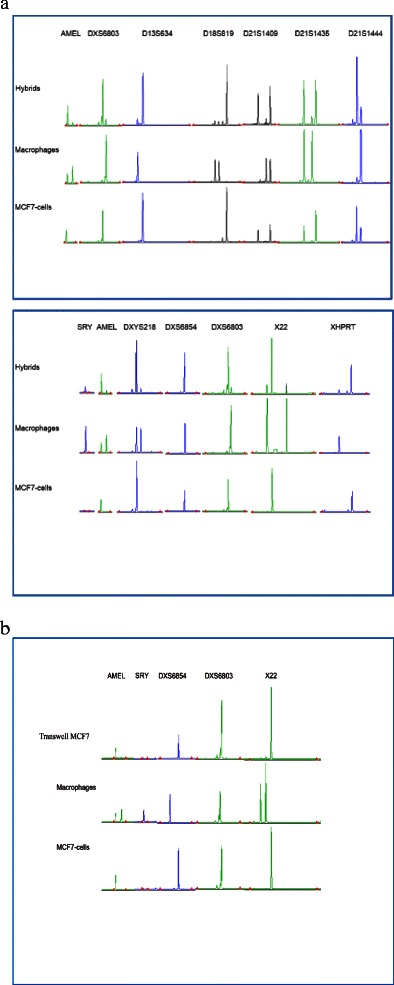
Electropherogram of STR analysis evaluates genetic expression in macrophages, MCF-7/GFP and macrophage/MCF-7 hybrid cells. MCF-7/macrophage hybrids were created by spontaneous fusion between M2 macrophages differentiated from monocytes harvested from male blood donors and GFP-labeled MCF-7 breast cancer cell. STR analysis with 24 markers on X and Y chromosomes and on chromosomes 13, 18 and 21 was used to determine the DNA profile and origin of the hybrid cells. The figure represents 12 of 17 shared loci detected in MCF-7/macrophage hybrids as a result of fusion between MCF-7 breast cancer cells and macrophages. The hybrids exhibit sex-determining region Y gene (SRY), which is an important indicator that these cells have arisen after fusion between macrophages and MCF-7 cells (**a**). MCF-7 cells from the transwell chamber system showed no shared STR loci confirming that niether cell fusion nor genetic exchange had occurred between the macrophages and MCF-7 cancer cells (**b**)

To confirm the origin of hybrids, we used short tandem repeats (STR) analysis, including loci on X and Y chromosomes as well as chromosomes 13, 18 and 21. The macrophages were M2-activated monocytes harvested from male blood donors and contained Y chromosome, whereas the MCF-7 cell line was derived from a female breast cancer patient and lacks the Y chromosome. Out of 24 markers found in macrophages and MCF-7 cancer cells, 17 were of the same allelic size in MCF-7/macrophage hybrids. For several loci, there was at least one allele common to macrophages and MCF-7 cancer cells (Table 1). These data represent genomic evidence that confirm the results from flow cytometry analysis and indicate that the hybrid cells originate from macrophages and the MCF-7 cancer cell line (Fig. 4a).

STR analysis of macrophages and MCF-7 cells grown in a transwell chamber system showed no shared STR loci. MCF-7 cells did not show any Y chromosome, confirming that cell fusion had not occurred between the macrophages and MCF-7 cancer cells in the transwell culture chamber system (Fig. 4b).

### Demographics of the MCF-7/macrophage hybrids

Approximately 2 % of hybrids were sorted after each MCF-7/macrophage hybridization experiment. The hybrids were isolated and cultured for several weeks. We observed that the proliferation rate of the hybrids was slower than that of the parent MCF-7 cells.

### Immunohistochemistry and CD163 expression levels in patient material

**Fig. 5:**
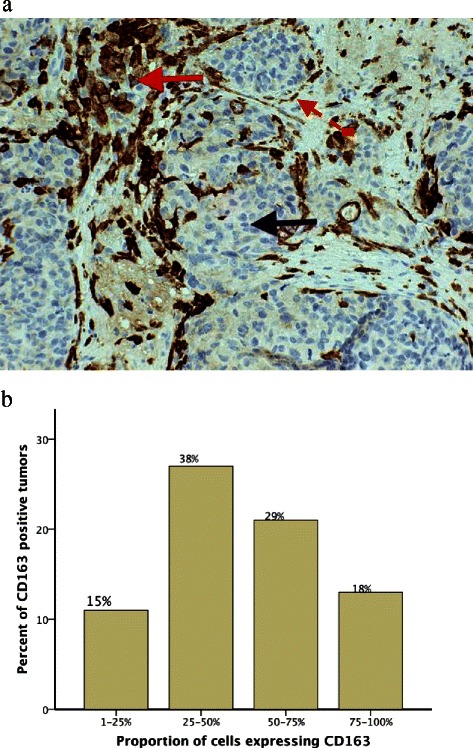
Immunostaining of breast cancer tissue sections. Serial breast tumor sections of 5 μm were stained with macrophage-specific antigen CD163 (Novocastra CD163, clone 10D6, mouse anti-human monoclonal antibody). (**a**) The histological picture of breast cancer (magnification of × 200). CD163-positive cells were pleomorphic with large nuclei and showed considerable heterogeneity in the distribution of CD 163 expression in different regions of the same section and in the same tumor specimens. CD163-positive cancer cells were organized in a growth pattern of clonal collections (red arrow). CD163-negative cancer cells (blue arrow) show similar morphological pattern but different phenotype (lacking macrophage phenotype). CD163 positive cancer cells can be distinguished morphologically from tumor associated macrophages. (red interrupted arrow) Note that macrophage nuclei are small and regular, whereas the cancer cells are enlarged and atypical with pleomorphic nuclei. and decreased cytoplasmic - nuclear ratio. (**b**) CD163 was expressed in 57 % of breast tumors. The proportion of CD163-positive cancer cells was greater than 50 % in 34 (47 %) of total 72 positive tumors

To evaluate the frequency of CD163 expression in clinical tumor material, breast cancer specimens from 127 women were used. CD163 staining was scored on a 5-tiered score as follows: 0 %, 1–25 %, 26–50 %, 51–75 %, and 76–100 % of the cancer cells. CD163 was expressed in 72 (57 %) patients. The cancer cells were considerable heterogeneous in the distribution of CD163 expression in different regions of the same section and in the same tumor specimen. CD163 positive cancer cells were organized in a growth pattern of one or more groups or clonal collections (Fig. 5a). The proportion of CD163 positive cancer cells was greater than 50 % in 34 (47 %) of the total of 72 positive tumors (Fig. 5b).

**Fig. 6:**
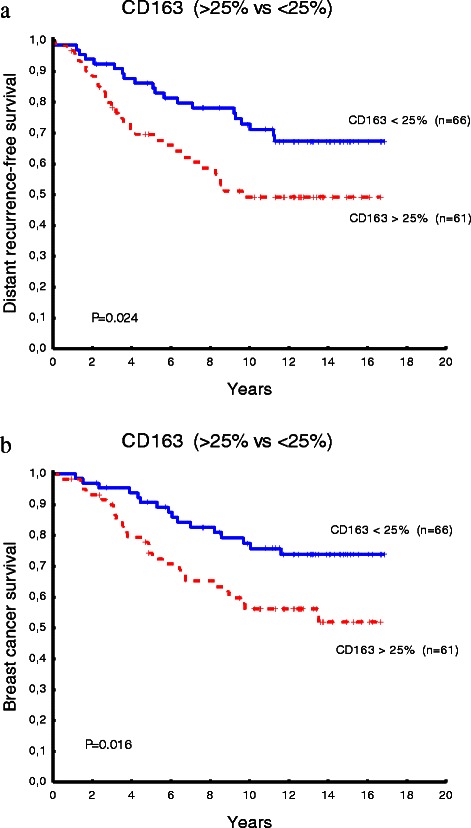
Expression levels of CD163 positive cancer cells in breast tumor section from 127 patients. The expression level with a cutoff point of 25 % of CD163 positive cancer cells in breast tumor sections in association to distant recurrence free survival (**a**) and disease free survival (**b**)

Based on the proportions of cancer cells expressing CD163, the previously mentioned expression scores were re-evaluated as cutoff points in assessment of CD163 expression as a marker in relation to survival data. Patients with breast tumors expressing CD163 in >25 % of cancer cells had significantly shorter survival time than patients with tumors expressing CD163 in <25 % of cancer cells, which was also reported on in an earlier study [11] (Fig. 6).

## Discussion

The genesis of CD163 expression as a macrophage trait in cancer cells reported in previous clinicohistopathological studies is unclear. It is proposed to be caused by fusion between macrophages and cancer cells. Paracrine cellular interaction in the tumor microenvironment has been suggested as an alternative explanation of macrophage traits in cancer cells. In the present study, macrophage traits in MCF-7 cancer cells are only generated by fusion with macrophages, proving they are not induced by cellular interaction between the macrophages and cancer cells.

Many reports present evidence that fusion between cancer cells and BMDCs, both in vivo and in vitro, may occur in cancer [7, 16, 17], but evidence of cell fusion and its clinical significance in human cancer remains controversial. Fusion events in human cancers are difficult to detect in a clinical context due to the lack of clinically safe tracing methods. The expression of tissue-specific markers, such as macrophage-specific antigen CD163, by cancer cells can be a reliable means of detecting the presence and significance of fusion in tumor tissue from clinical patient material. Several clinicopathological studies reported CD163 expression by cancer cells in breast tumors [11, 14], colorectal [10], and urinary bladder cancers [18]. CD163 expression was associated with advanced tumor stages and poor prognosis. However, these observations in cancer cell phenotype can be caused by other mechanisms, such as intercellular genetic exchange and paracrine interaction [19].

Transwell experimental in vitro models are well established methods of investigating cellular interaction. Such models have been used to show that breast cancer cells alter the nature of their surrounding cells, such as fibroblasts and macrophages, to support their own progression through paracrine signaling [20, 21]. Yang et al. reported that macrophages stimulated by IL-4 regulated the invasiveness of breast cancer cells through exosome-mediated delivery of the oncogenic miR-223 [22]. In this study, the MCF-7 cancer cells did not acquire macrophage phenotype by in vitro interaction with macrophages. MCF-7 cancer cells obtained CD163 and CD45 expression only by hybridization between MCF-7 cancer cells and macrophages. These findings indicate that CD163 expression in cancer cells can be used as a surrogate marker to detect cell fusion generally in human solid tumors, and specifically in breast cancer.

Cell fusion is a common biological process that produces viable cells and plays a major role in mammalian development and differentiation [23]. Spontaneous cancer-stromal cell fusion is a rare, but active, stepwise process that requires the participation of both cell types [24]. In the present study, the hybrids were generated spontaneously at an average rate of 2 % and were able to survive cultured in RPMI 1640 medium for several weeks. Thus, although the proportion of hybrids may be small in relation to the total tumor mass, the spontaneity of cell fusion, and the survival and growth of the hybrids may cause the development of derivative clones that might have important clinical implications. It has been postulated that 1 gram of tumor mass contains approximately 1 x 10^8^ tumor cells [25, 26]. Based on this calculation, the hybrid rate of 2 % means that each gram of cancer may contain approximately 2 million hybrids. This observation is consistent with the fact that tumor size is a prognostic factors in breast cancer [27]. Furthermore, fusion efficiency can be proportional to the malignant level of tumor cells [28]. In this study, the proportions of CD163 positive cancer cells were not associated to survival rates. On the other hand, a cutoff point of >25 % was significantly related to both disease free and recurrence free survival. These data indicate that CD163 might be useful in clinical context as histopathological marker for detection of fusion between macrophages and tumor cells in breast cancer.

Cancer is a Darwinian adaptive system where rare genetically unstable cells thwart biological selective pressure [29]. Clonal expansion is traditionally thought to be driven by genetic and epigenetic changes inherited by cell division [30]. Cell-fusion-mediated nuclear reprogramming results in genetic and epigenetic alterations [31]. The histopathological analysis in this study clearly shows that CD163-positive cancer cells are organized in a growth pattern of one or more collections. Thus, tumor cells with macrophage traits may acquire competitive advantages over the cells in tumor stroma. In the light of these observations and previous arguments, we believe that cell fusion might contribute to clonal expansion and the heterogeneity of cancer cells.

## Conclusions

Macrophage traits, represented by CD163 and CD45 expression in cancer cells, are due to fusion between cancer cells and macrophages, and cannot be explained by cellular interaction between these cells. Cell fusion might contribute to clonal expansion of cancer and generate considerable numbers of hybrids in tumor stroma. The cutoff point >25 % of tumor cells expressing CD163 in tumor samples is correlated to DSS and DRFS rates suggesting that CD163 might be useful as macrophage/cancer cell fusion marker in clinical context.
